# Revised Control Barrier Function with Sensing of Threats from Relative Velocity Between Humans and Mobile Robots

**DOI:** 10.3390/s25134005

**Published:** 2025-06-27

**Authors:** Zihan Zeng, Silu Chen, Xiangjie Kong, Xiaojuan Li, Chi Zhang, Guilin Yang

**Affiliations:** 1 School of Mechanical Engineering, Xinjiang University, Urumqi 830047, China; 2 Ningbo Institute of Materials Technology and Engineering, Chinese Academy of Sciences, Ningbo 315201, China

**Keywords:** mobile robot, human–robot interaction, safety constraint, control barrier function, relative velocity, obstacle avoidance

## Abstract

The mobile robot, which comprises a mobile platform and a robotic arm, has been widely adopted in industrial automation. Existing safe control methods with real-time trajectory alternation face difficulties in efficiently identifying threats from fast relative motion between humans and robots, causing hazards in environments of dense human–robot coexistence. This work firstly builds a safe mobile robot control framework in the kinematic sense. Secondly, the proximity between parts of a human and a mobile robot is efficiently solved by convex programming with parametric description of skew line segments. It is also no longer required to perform case-by-case analysis of skew line segments’ relative pose in space. Thirdly, a novel threatening index is proposed to select the most threatened human parts based on mutual projection of human–robot relative velocity and their common normal vector. Eventually, this index is incorporated into the safety constraint, showing the improved safe control performance in the simulated human–mobile robot coexistence scenario.

## 1. Introduction

A mobile robot incorporates a mobile platform and a robotic arm to form a hig y flexible and efficient production unit [1]. This integration offers innovative solutions for managing complex tasks [2]. While human–robot collaboration scenarios continue to proliferate, significant challenges remain in establishing reliable safe control frameworks for dynamic environments [3].

Many methods have been proposed to address the challenges of path planning for mobile robots in complex environments, such as potential field [4,5] and danger field-based methods [6]. These methods are widely used for real-time obstacle avoidance due to their high computational efficiency. Control barrier functions (CBFs) are defined in control systems by ensuring that a system’s state remains within a predefined safe state set through the design of constrained control inputs [7]. It is particularly efficient in real-time obstacle avoidance if the safe conditions are set up as inequality constraints in quadratic programming. However, CBFs may face issues of high computational complexity and occasional collision with high-dimensional space or low-sampling-rate perception and control, and their definition of obstacle distances may not be comprehensive enough [8]. In recent years,  deep reinforcement learning (DRL) [9], neural belief tracking (NBT) [10], and other methods based on artificial intelligence have also been applied to trajectory planning in the human–machine interaction. By learning and predicting the trajectories of dynamic obstacles, neural networks can significantly enhance the intelligence and adaptability of path planning [11,12]. However, they lack formal safety guarantees, and their performance relies on high-quality training data [13,14]. The CBF-based safe reinforcement learning (CSRL) algorithm has been employed to address target pursuit problems [15]. The combination of CBF and model predictive control (MPC) can address the efficiency issues of online obstacle avoidance in unstructured environments filled with static and dynamic obstacles [16]. The path planning and task allocation in multi-robot systems has recently been optimized by integration of DRL, MPC, and graph neural networks (GNNs) [17]. However, alternating the trajectory in the real time remains a problem when these advanced networks are incorporated.

By sensing and adjustment of the distance from the obstacles, robots can prevent collisions [18]. The trajectory of mobile robots in the environment with static obstacles can be planned through offline programming methods [19]. For a robotic arm with a fixed base, a conservative approach is to define the safe boundary based on robotic arm’s own workspace [18]. However, mobile robots sometimes need to deal with crowded, dynamic human–robot environments, where the uncertainty of human motion greatly challenges the real-time trajectory planning [20]. The difficulty of trajectory planning is further increased with mobile robots as the kinematics of the robotic arms couples with that of the mobile platform, especially when dealing with multiple moving humans or obstacles [21]. The determination of minimum human–robot distance is done by case-by-case analysis depending on the relative locations of the skew line segments and their common normals [22]. The “look-backward-and-forward” algorithm is further developed to predict the human trajectory [23], while the estimation of prediction error is further incorporated into the discrete-time control barrier function (DCBF) constraints. However, for crowded environment with dense and fast-moving humans and mobile robots, considering only the minimum distance between the individual parts of a human and a mobile robot is insufficient to indicate the threat in the future. Notably, the human-robot relative velocity critically indicates the level of danger [24] or injury severity [25] when facing potential impacts. They further affect operators’ mental workload, anxiety, and risk perception [26]. The robot’s velocity is included as a sigmoid-like smooth function term to shape the CBF [27]. A broader range of such constraints are included, such as robot joint velocities and the end-effector’s velocity, as well as wall boundaries and cylindrical capsules for human and robot parts [28]. However, how to incorporate the relative velocity into the safety index is not well studied when designing the robot controller.

This paper aims to incorporate the human–robot relative velocity in the control barrier function (CBF), to achieve safe control with real-time trajectory alternation for mobile robots in human–robot coexisting environments. The main contributions are as follows:

1. The coupling kinematics of the mobile robot with a mobile platform and a robotic arm is formed in the kinematic sense rather than solely with the fixed-base robot arm in [22], so that the kinematic control problem in human–robot environments is set up with a discrete-time control barrier function (DCBF) and discrete-time control Lyapunov function (DCLF).
2. By setting up the parametric description of skew line segments, the minimum distance between a pair of human skeletons and a link of a robotic arm (or an outline of a mobile platform) is efficiently solved in real time by convex programming. Compared with [22,28], it is no longer necessary to make a case-by-case analysis depending on the relative locations of the skew line segments and their common normal.
3. By mutual projections of relative velocity of parts of a human and a mobile robot and their common normal vector, two projection indexes are given. Thereafter, a novel threat index is formed to give a normalized “distance” to select the most threatened human parts. In this way, the relative velocities between parts of humans and mobile robots are successfully incorporated into the DCBF-based constraint rather than just their absolute velocities [27,28].

Simulation of human and mobile-robot environments validates the effectiveness of the proposed safe control method for real-time trajectory alternation.

## 2. Related Works

Control Barrier Functions (CBFs) have emerged as a formal methodology for guaranteeing system safety in automation and robotics. They construct dynamic safety boundaries by transforming complex safety constraints into computable mathematical conditions, embedding these conditions within controller optimization frameworks to ensure system states remain within safe parameters [29]. Compared to traditional approaches, CBFs uniquely provide rigorous mathematical safety guarantees while meeting the real-time demands of modern control systems. Key research contributions include:

*Standard CBFs based on Zeroing Barrier Functions*: This class of CBFs define safe sets via differential inequalities governing system trajectories to prevent boundary violations [7]. Applications include collision avoidance for autonomous vehicles, collaborative robots, and UAV formations. Representative implementations encompass event-triggered CBFs for efficient traffic control [30], dual-arm coordination with obstacle avoidance [31], and minimum-intervention collision-free UAV control [32].

*High-Order CBFs*: This class of CBFs employ recursively defined function chains to convert safety constraints with relative degrees greater than one into feasible conditions explicitly incorporating control inputs [33]. They have been applied to predictive human–robot safety interaction and fall prevention for legged robots. Notable examples include control of articulated robots using dynamic obstacle trajectory prediction [22] and dynamic equilibrium maintenance combined with obstacle-aware trajectory optimization for bipedal robots [34].

Current challenges involve enhancing adaptability and robustness under strong dynamic coupling and unmodeled disturbances [35], alleviating computational bottlenecks (e.g., quadratic programming for multi-agent systems) [36], and mitigating prediction error accumulation in dynamic environments [37]. Potential solutions include cross-disciplinary integration, such as incorporating reinforcement learning into stochastic control frameworks [38]. In view of the above, CBFs are still promoted as a universal design paradigm for safety-critical applications, enabling reliable unmanned systems in industrial automation [39] and medical surgery [40].

## 3. Problem Formulation

This section firstly models the mobile robot under a discrete-time kinematic control framework. Later, the existing safe control framework is imported with both discrete-time control barrier function (DCBF) and discrete-time control Lyapunov function (DCLF).

### 3.1. Control of a Mobile Robot

**Figure 1:**
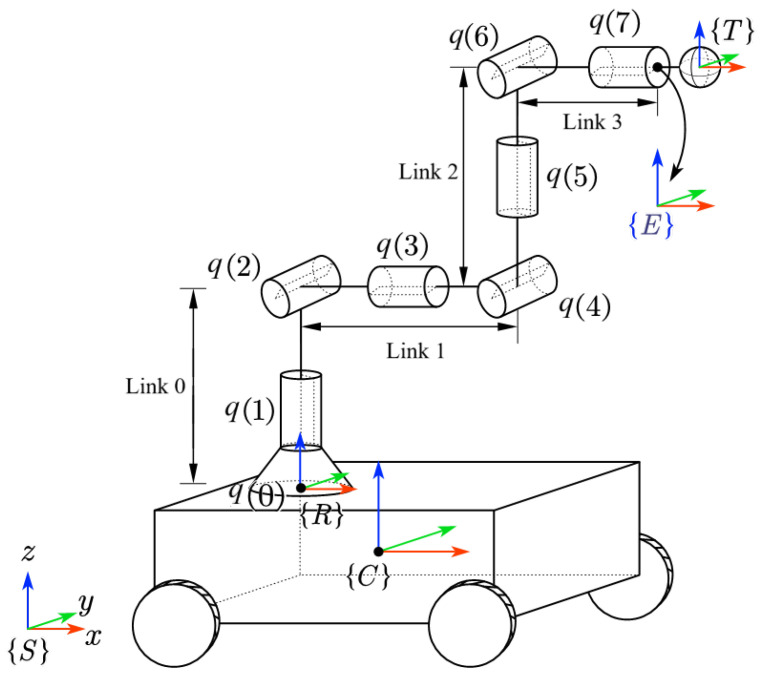
Schematics of a mobile robot.

The mobile robot consists of a mobile platform and a robotic arm, as shown in Figure 1.

The mobile platform is equipped with four Mecanum wheels, and its kinematics are given as:

(1) $$\begin{array}{ccc} p_{k+1} & = & p_{k}+v_{k}\delta_{t} \end{array}$$

(2) $$\begin{array}{ccc} v_{k} & = & J_{c}u_{k} \end{array}$$

where the subscript *k* denotes the *k*-th instant for all variables, $p\in R^{3}$ represents the pose of mobile platform, $v\in R^{3}$ is its planar velocity, $u\in R^{4}$ is its control input and $J_{c}\in R^{3\times 4}$ is its Jacobian matrix.

Assume a robotic arm with *n* revolute joints is installed on the mobile platform, and its joint displacements are denoted as $q\in Q\subseteq R^{n}$. Hence, its kinematics are given by

(3) $$\begin{array}{ccc} q_{k+1} & = & q_{k}+w_{k}\delta_{t} \end{array}$$

(4) $$\begin{array}{ccc} a_{k} & = & g\left(\chi_{k}\right)={\hat{\bar{p}}}_{k}\;T_{R}^{C}\;T_{a}\left(q_{k}\right)\in SE\left(3\right) \end{array}$$

where $\chi ={\left[\begin{array}{cc} q^{T} & p^{T} \end{array}\right]}^{T}$, $\bar{p}={\left[\begin{array}{cccccc} p(1) & p(2) & 0 & 0 & 0 & p(3) \end{array}\right]}^{T}$, *a* is the pose in the task space, $T_{a}\left(q\right)$ is the forward kinematics of the robotic arm, $T_{R}^{C}$ is the homogeneous transformation matrix (HTM) from the frame of the mobile platform to the base frame of the robotic arm, and *w* is the control input of the robotic arm, “∧” is the operator for $\left(•\right)\in R^{6}\to (\hat{•})\in SE\left(3\right)$.

The task is to track a desired pose $a_{d}$ with the mobile robot’s end effector. We can design a discrete-time feedback controller *s* with a control gain $K_{s}$:

(5) $$s_{k}=K_{s}{\left[{\left(a_{k}\right)}^{-1}a_{d,k}\right]}^{\vee }$$

where *s* is the velocity of the end effector, “∨” is the operator for $\left(•\right)\in SE\left(3\right)\to {\left(•\right)}^{\vee }\in R^{6}$, and 

(6) $$s_{k}=J_{e,k}\nu_{k}≜\left[\begin{array}{cc} J_{\mathrm{base},k} & J_{a,k} \end{array}\right]\left[\begin{array}{c} u_{k}\\ w_{k} \end{array}\right]$$

In (6), $J_{a}\in R^{6\times n}$ is the Jacobian matrix of the robotic arm, $J_{\mathrm{base}}=\left[{\mathrm{Ad}}_{T_{a}^{-1}\left(q\right)\;{\hat{\bar{p}}}^{-1}}\right]{\bar{J}}_{c}$, where $\left[{\mathrm{Ad}}_{\left(•\right)}\right]$ is adjoint transformation. In addition,

$${\bar{J}}_{c}=\left[\begin{array}{c} J_{c}\left(1,•\right)\\ J_{c}\left(2,•\right)\\ 0_{1\times 4}\\ 0_{1\times 4}\\ 0_{1\times 4}\\ J_{c}\left(3,•\right) \end{array}\right]\in R^{6\times 4}.$$

where $J_{c}\left(1,•\right)$ is the first row of $J_{c}$, etc. So the velocity (i.e., the control signal) of the entire mobile robot in the joint space $\nu_{k}$ is:

(7) $$\nu_{k}=J_{e,k}^{†}K_{s}{\left[{\left(a_{k}\right)}^{-1}a_{d,k}\right]}^{\vee }≜w\left(\chi_{k}\right)$$

where $J_{e}^{†}$ is the (pseudo-)inverse of $J_{e}$.

### 3.2. Safety and Stability Constraints

To achieve safe human–robot interaction, a safe set *C* is defined through a continuous differentiable function H:Q→R.

(8) $$C=\{\chi_{k}\in Q:B\left(\chi_{k}\right)\geq 0\}$$

where B(·) is a discrete-time control barrier function (DCBF). $\Delta B\left(\chi_{k}\right)=B\left(\chi_{k+1}\right)-B\left(\chi_{k}\right)\neq 0$, χ∈∂C and

(9) $$\Delta B\left(\chi_{k}\right)\geq -\gamma B\left(\chi_{k}\right)$$

with a constant coefficient γ∈(0,1]. This ensures the safe operation of the system.

Further, the stability of the system is constrained by the discrete-time Lyapunov function (DCLF) $V(\chi_{k})$, and 

(10) $$\Delta V\left(\chi_{k}\right)\leq -\alpha V\left(\chi_{k}\right)+\delta$$

where $\Delta V\left(\chi_{k}\right)=V\left(\chi_{k+1}\right)-V\left(\chi_{k}\right)$, and α∈(0,1] is a constant, δ≥0 is a slack variable.

Based on the above constraints, the controller under human–robot coexisting environment is synthesized by:

(11) $$\begin{array}{cc} \nu_{k}^{*}=\arg\min(∥\nu_{k}-{\bar{\nu }}_{k}{∥}^{2} & +{\beta \delta }^{2}) \end{array}$$

(12) $$\begin{array}{cc} s.t.\;\Delta B(\chi_{k},\nu \left(\chi_{k}\right)) & +\;\gamma B(\chi_{k})\geq 0 \end{array}$$

(13) $$\begin{array}{cc} \Delta V(\chi_{k},\nu \left(\chi_{k}\right)) & +\;\alpha V(\chi_{k})\leq \delta \end{array}$$

${\bar{\nu }}_{k}={\left[\begin{array}{cc} {\bar{u}}_{k}^{T} & {\bar{w}}_{k}^{T} \end{array}\right]}^{T}$ are the desired velocity when there is no human interruption, β>0 is the slack variable.

## 4. Main Results

### 4.1. Distance Between a Mobile Robot and Human

This subsection aims to effectively seek the minimum distance between a pair of skew line segments by forming a convex optimization problem with parametric description of the points on the line segments.

**Figure 2:**
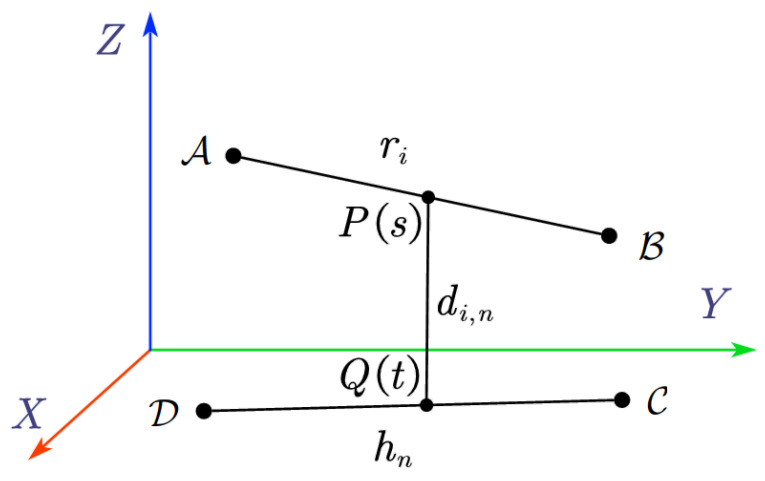
Distance between a pair of skew lines.

Obtaining the closest distance between humans and the mobile robots in real time is necessary to establish CBF constraints in (12) for safe human–robot interaction. To calculate their closest distance, one can model the human body as a skeletal structure and simplify the robotic arm as a linkage mechanism, while the moving platform is modeled as a rectangle with four enclosed line-segments. At this point, the problem is transformed to calculation of the distance between spatial line segments. Taking Figure 2 as an example, let points A and B be the endpoints of the *i*-th link of the robotic arm or outline of the moving platform, and points C and D be the endpoints of the *n*-th segment of human skeleton. The parametric equations of the pair of skew line segments (i,n) are:

(14) $$\begin{array}{c} P(s)=A+s(B-A),\;s\in [0,1] \end{array}$$

(15) $$\begin{array}{c} Q(t)=C+t(D-C),\;t\in [0,1] \end{array}$$

where *s* and *t* are normalized distances from points A and C, respectively.

The problem is transformed into finding points P(s) and Q(t) such that the distance between them is minimized. This is achieved by the following convex optimization problem:

(16) $$\min f_{i,n}\left(s,t\right)\;s.t.\;s,t\in \left[0,1\right]$$

where

(17) $$\begin{array}{ccc} f_{i,n}\left(s,t\right) & = & {∥P\left(s\right)-Q\left(t\right)∥}^{2}\\ & = & ∥\left(A+s\left(\vec{AB}\right)\right)-\left(C+t\left(\vec{CD}\right)\right){∥}^{2} \end{array}$$

To deal with the constraints, we construct the Lagrangian function:

(18) $$L=f_{i,n}\left(s,t\right)-\lambda_{1}s+\lambda_{2}\left(s-1\right)-\lambda_{3}t+\lambda_{4}\left(t-1\right)$$

where Lagrange multipliers $\lambda_{1},\ldots ,\lambda_{4}$ are all non-negative. The solution $\left(s^{*},t^{*}\right)$ to the above optimization problem needs to satisfy the Karush–Kuhn–Tucker (KKT) conditions:

(19) $$\begin{array}{ccc} \frac{\partial L}{\partial s} & = & 2{\left(\vec{AB}\right)}^{T}\left(\vec{CA}+s\left(\vec{AB}\right)-t\left(\vec{CD}\right)\right)\\ & & +\lambda_{2}-\lambda_{1}=0 \end{array}$$

(20) $$\begin{array}{ccc} \frac{\partial L}{\partial t} & = & -2{\left(\vec{CD}\right)}^{T}\left(\vec{CA}+s\left(\vec{AB}\right)-t\left(\vec{CD}\right)\right)\\ & & +\lambda_{4}-\lambda_{3}=0 \end{array}$$

(21) $$\begin{array}{ccc} s,t & \in & \left[0,1\right] \end{array}$$

(22) $$\begin{array}{ccc} \lambda_{i} & \geq & 0 \end{array}$$

(23) $$\begin{array}{ccc} \lambda_{1}s & = & \lambda_{2}\left(s-1\right)=\lambda_{3}t=\lambda_{4}\left(t-1\right)=0 \end{array}$$

Thus, the closest distance between all pairs of skew line segments is calculated by

(24) $$d^{\min}=\underset{i,n}{\inf}\sqrt{f_{i,n}^{*}}$$

where $f_{i,n}^{*}$ is optimal $f_{i,n}$ obtained by solving (16) with the *i*-th line segment of the mobile robot and *n*-th segment of the human skeletons.

### 4.2. The Threatening Index Based on Relative Velocity

Based on the minimum distance between a human and a mobile robot, we can set up the corresponding safety constraint. However, this is insufficient to ensure the safety in real-time under complex interaction scenarios, especially when the humans and robots are moving fast. Thus, this subsection aims to extend the definition of “minimum distance” by considering the factor of the relative velocity between a human and a robot.

**Figure 3:**
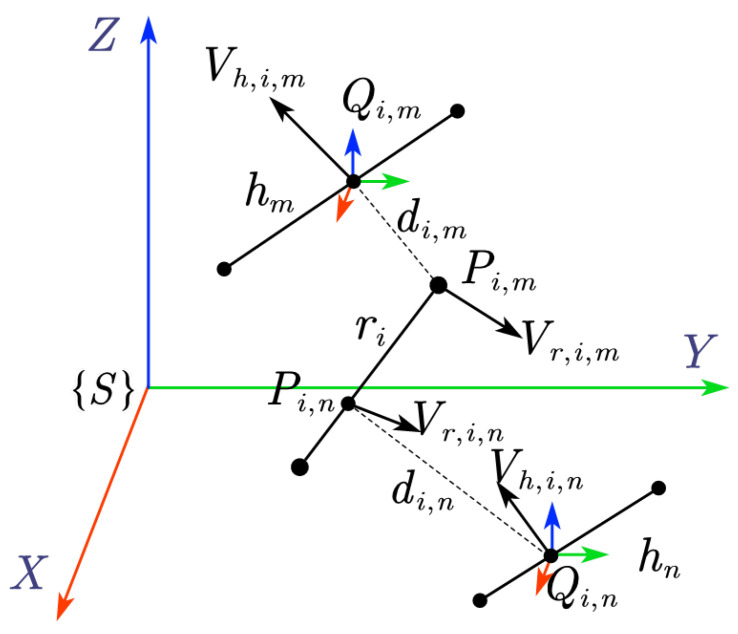
Minimum distance and relative velocity between the skew lines.

As shown in Figure 3, $r_{i}$ denotes the *i*-th link of the robot, $h_{m}$ and $h_{n}$ represent two segments of the human skeleton, and $V_{r,(•)}$ represents the velocity of $P_{\left(•\right)}$. $V_{h,(•)}$ represents the velocity of $Q_{\left(•\right)}$. According to the method proposed in Section 4.1, the closest points between $r_{i}$ and $h_{m}$ are $P_{i,m}$ and $Q_{i,m}$, while the closest points between $r_{i}$ and $h_{n}$ are $P_{i,n}$ and $Q_{i,n}$. It can be observed that $d_{i,m}<d_{i,n}$. However, point $Q_{i,m}$ is moving away from point $P_{i,m}$, while point $Q_{i,n}$ is moving towards point $P_{i,n}$. Under the proximity-based DCBF constraint (12), $d_{i,m}$ is taken as the minimum distance, at which the robot will take evasive action towards point $Q_{i,m}$. However, this ignores the more threatening point $Q_{i,n}$, which may lead to a collision with $h_{n}$ in the near future. Therefore, when constructing the DCBF constraint, it is also necessary to consider the units that give greater threats in relative motion in addition to the minimum distance between humans and the robot.

**Figure 4:**
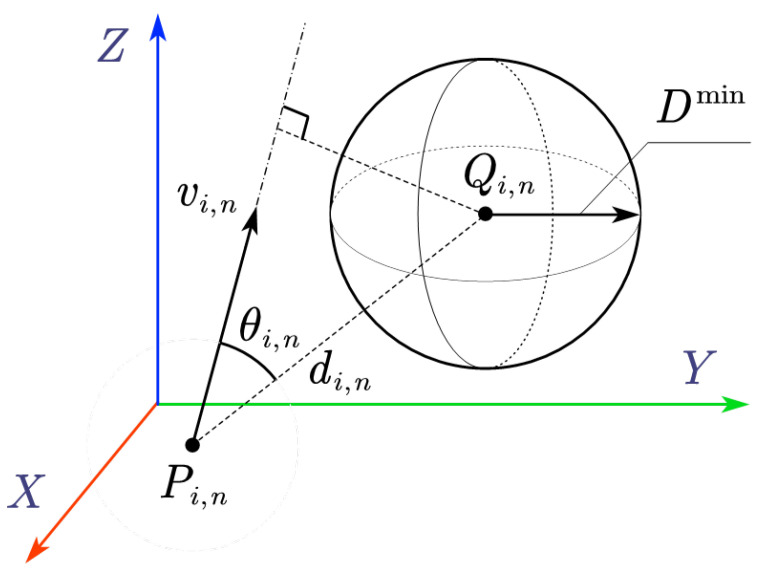
The influence of relative velocity to the safe threat.

Taking $r_{i}$ and $h_{n}$ in Figure 3 as an example, the threat level to the system is quantified by the relative velocities between humans and robots. This is given by the threatening index based on the principle illustrated in Figure 4.

As shown in Figure 4, $v_{i,n}$ is the relative velocity between $P_{i,n}$ and $Q_{i,n}$ and

$$v_{i,n}=V_{r,i,n}-V_{h,i,n}$$

θ is the angle between the relative velocity vector and the distance vector (sharing a common starting point), and $D^{\min}$ is the predefined threshold of safe distance between humans and robots. Hence, the threatening index $k_{i,n}$ for skew line segment pair (i,n) is calculated as

(25) $$\begin{array}{ccc} k_{I,i,n} & = & \frac{v_{i,n}\;\delta_{t}}{d_{i,n}\cos\theta_{i,n}} \end{array}$$

(26) $$\begin{array}{ccc} k_{A,i,n} & = & \frac{D^{\min}-d_{i,n}\sin\theta_{i,n}}{D^{\min}} \end{array}$$

(27) $$\begin{array}{ccc} k_{i,n} & = & \left\{\begin{array}{c} 0,\;\mathrm{if}\;\;k_{A,i,n}<0\\ k_{I,i,n}\left(k_{A,i,n}+1\right),\;\mathrm{if}\;k_{A,i,n}\geq 0 \end{array}\right. \end{array}$$

where $k_{I,i,n}$ is related to the intensity of the robot’s impact on the human body at the current relative velocity. $k_{A,i,n}$ is related to the angle of impact at the time of collision, and $\delta_{t}$ is the control period.

- When $k_{A,i,n}<0$, it indicates that the robot moving at the current relative velocity will not intrude into the safety range of the obstacle. Thus, the threatening index is set to zero.
- When $k_{A,i,n}\geq 0$, the robot has the risk of intruding into the safety range of the obstacle at the current relative velocity. In this case, $k_{i,n}$ is set to $k_{I,i,n}\left(k_{A,i,n}+1\right)$, where $\left(k_{A,i,n}+1\right)$ ensures that the threat coefficient is at least $k_{I,i,n}$ when $k_{A,i,n}=0$. Thus, a non-zero threatening index is issued.

We compute the threatening index $k_{i,n}$ between each pair (i,n) and select the maximum $\bar{k}$. Later, we obtain the minimum $\underline{d}$ across distance $f_{i,n}$ for pair (i,n) with the identical maximum threatening index $\bar{k}$:

(28) $$\begin{array}{ccc} \bar{k} & = & \max_{i,n}k_{i,n}, \end{array}$$

(29) $$\begin{array}{ccc} \underline{d} & = & \min\{f_{i,n}|k_{i,n}=\bar{k}\} \end{array}$$

We can further refine the selection of the object pairs (i,n), all of which have $f_{i,n}=d^{\min}$. This is done by selecting the pair with the highest threatening index:

(30) $$\begin{array}{ccc} S & = & \{\left(i,n\right)|f_{i,n}=d^{\min}\}, \end{array}$$

(31) $$\begin{array}{ccc} \left(i^{*},n^{*}\right) & = & \arg\max_{(i,n)\in S}k_{i,n}, \end{array}$$

### 4.3. Revised Safety Constraints

After identifying the most threatened human parts with the threatening index based on human–robot relative velocity, a more comprehensive safety constraint is constructed in this section.

Based on the distance and threatening index between humans and robots, we can design multi-objective constraints for the safe set C defined in (12) with the prescribed safe distance $D^{\min}$ as

(32) $$\Delta B\left(\chi_{k}\right)=\lambda_{D}B_{D}+\left(1-\lambda_{D}\right)B_{V}$$

where $B_{D}$ corresponds to the constraint of the closest distance, and $B_{V}$ corresponds to the distance constraint of the object with the maximum threatening index based on the relative velocity. The specific constraints are as follows:

(33) $$\begin{array}{ccc} B_{D} & = & d^{\min}-D^{\min}\geq 0 \end{array}$$

(34) $$\begin{array}{ccc} B_{V} & = & \underline{d}-D^{\min}\geq 0 \end{array}$$

$\lambda_{D}$ is the weight distribution coefficients for the two constraints, allocated based on the threatening index:

(35) $$\lambda_{D}=\frac{1}{\bar{k}+1}$$

To ensure the forward invariance of the set C, the DCBF-based constraints (12) are further given as:

(36) $$\begin{array}{ccc} d_{k+1}^{\min}-d_{k}^{\min} & \geq & -\gamma (d_{k}^{\min}-D^{\min}) \end{array}$$

(37) $$\begin{array}{ccc} {\underline{d}}_{k+1}-{\underline{d}}_{k} & \geq & -\gamma ({\underline{d}}_{k}-D^{\min}) \end{array}$$

Unlike prior studies on fixed-base robotic arms that only consider the distance between humans and the robotic arm [22], this composite constraint considers both human–robot distance and relative velocity.

When the mobile robot aims to reach a desired posture after completing the trajectory, we can construct a constraint about $V(\chi_{k})$ as

(38) $$\begin{array}{ccc} d_{g,k} & = & ∥{℘}_{k}-{℘}_{d,k}{∥}_{2} \end{array}$$

(39) $$\begin{array}{ccc} V(\chi_{k}) & ≜ & \epsilon -d_{g,k}>0 \end{array}$$

where $d_{g}$ is the distance between the current end-effector position *℘* and the desired position ${℘}_{d}$. *℘* and ${℘}_{d}$ are the position vectors extracted from the *a* in (4) and $a_{d}$ in (5), respectively. ϵ is a very small constant. So the DCLF-based constraint in (13) is given as:

(40) $$-d_{g,k+1}+d_{g,k}\leq -\alpha \left(\epsilon -d_{g,k}\right)+\delta$$

**Figure 5:**
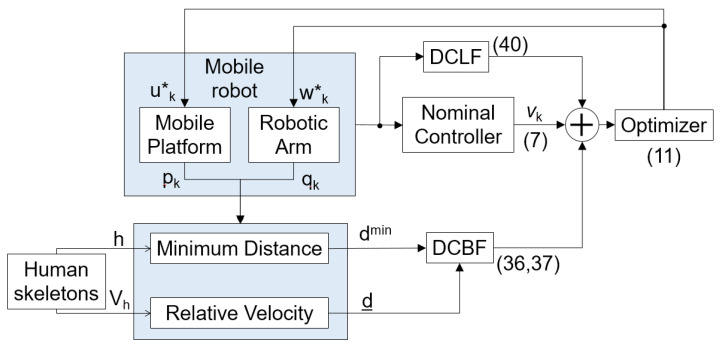
The overview of proposed safe control method.

By constructing the aforementioned constraints, the mobile robot can be equipped with a framework based on the hybrid DCBF and DCLF, ensuring safe and efficient operation in dense human-robot coexistence environments. The proposed safe control method for a mobile robot is summarized in Figure 5.

## 5. Simulation

### 5.1. The Setting of Simulation

To verify the effectiveness of the proposed method, two mobile robots are imported for simulation. Each consists of a mobile platform with four Mecanum wheels and a KUKA iiwa robotic arm. The task is to let them approach their corresponding target poses in a human–robot environment, interrupted by three humans. The paths of the humans are manually preset but not known in advance by the mobile robots, and the posture data of human bodies are obtained from a motion capture dataset. The simulation is performed on a workstation equipped with a 12th Gen Intel® Core™ i9-12900H 2.50 GHz processor. The optimization problem is solved by fmincon function in MATLAB(2022a), selecting the interior-point method for its low memory footprint and real-time capability. The initial solution is set to the current state values.

**Figure 6:**
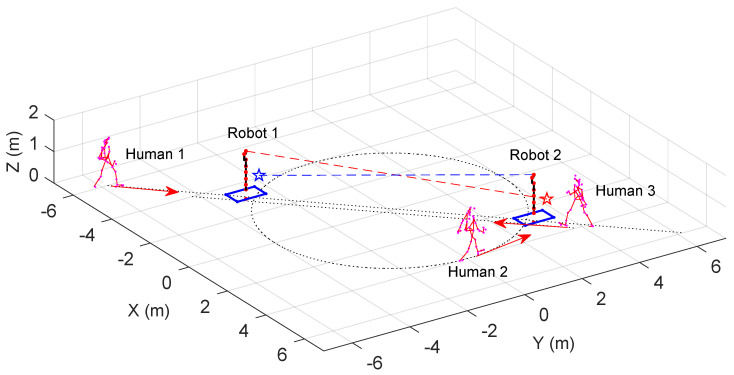
The initial state in the human–robot coexistence environment.

Figure 6 illustrates the initial state of the mobile robot. A red dot in the XY-plane marks the origin of the mobile platform’s frame, and the blue lines outline its edges. A robotic arm is mounted on each mobile platform. The joint positions are indicated by red dots, and the links are depicted as black line segments. A pentagram denotes the target for the robotic arm’s end-effector. The mobile robots aim to reach their targets while maintaining safe distances from all humans. Robot 1’s mobile platform starts at ${\left[-3,-3,\pi /2\right]}^{T}\in SE\left(2\right)$, and its arm is initially rested vertically. The goal is to let the end effector move to ${\left[0,0.97,0,3.68,3,1\right]}^{T}\in SE\left(3\right)$. Robot 2’s platform starts at ${\left[3,3,-\pi /2\right]}^{T}\in SE\left(2\right)$ with its arm being initialized in the same way as Robot 1, targeting ${\left[0,0.97,0,-2.32,-3,1\right]}^{T}\in SE\left(3\right)$. The homogeneous transformation matrix from the mobile platform’s frame to the robotic arm base is ${\left(T_{R}^{C}\right)}^{\vee }={\left[0,0,0,0,0,0.3\right]}^{T}$. Human 1 moves back and forth between the robots’ target points, Human 2 orbits the origin, and Human 3 traverses radially across the mobile robots’ paths.

When screening obstacle-avoidance objects, the mobile platform is simplified as a planar quadrilateral bounding box. For the robotic arm, the rotations of joints 1, 3, and 5 in the KUKA iiwa configuration do not affect the positions of subsequent links. Therefore, the robotic arm can be simplified into four segments: from the base to joint 2 (Link 0 in Figure 1, joint 2 to joint 4 (Link 1), joint 4 to joint 6 (Link 2), and joint 6 to joint 7 (Link 3). Link 0 is fixed to the mobile platform and will not extend beyond the planar quadrilateral bounding box of the mobile platform. The mobile robot is thus simplified into four objects: the planar quadrilateral bounding box of the mobile platform and Link 1, 2, and 3 of the robotic arm.

By incorporating the DCBF-based constraints (36), (37) into the optimization formulation (12), we can obtain ${\dot{\chi }}_{k}$ to drive a mobile robot to maintain a safe distance from humans or other obstacles during movement. The DCBF constraints based on proximity and threatening index are set up for the mobile platform and Link 1, 2, and 3. Consequently, there are eight DCBF-based constraints to ensure safety in such human–robot coexistence environments. When the end-effector approaches the target point, the DCLF-based constraint (13) is applied to all control methods, ensuring that the end-effector moves as close as possible to $a_{d}$ if no human is interfering while maintaining stability. Consequently, the optimization problem (11) is formulated, and we impose the following joint limits for the mobile platform:

- Limits of wheels’ velocity: [10,10,10,10];
- Limits of joints’ velocity for the robotic arm: [85,85,100,75,130,135,135];
- Limits of joints’ position limits for the robotic arm: [170,120,170,120,170,120,175].

We would like to compare our safe control method with the conventional one containing DCBF with proximity-based DCBF. Further, we also compare our method with the Dynamic Window Approach (DWA) and Artificial Potential Field (APF) method to accomplish navigation and obstacle avoidance for the mobile platform and robotic arm, respectively [41]. For the method with proximity-based DCBF, the simulation setup is identical to that used for both proximity-based and threatening-index-based DCBF. For the DWA, the prediction horizon is set to six steps, and the scoring weights for the target, velocity, obstacles, orientation, and path smoothness are [0.70, 0.15, 0.05, 0.05, 0.05]. For the APF, the obstacle repulsion and the target attraction coefficients are set to be 0.5 and 1.5 accordingly. The codes for implementation of this simulation example are given in the Supplementary Material.

### 5.2. Results and Discussions

**Figure 7:**
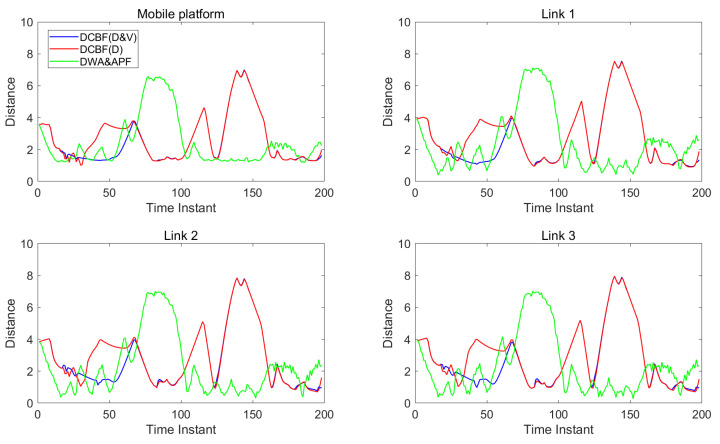
The distance (in meters) between humans and the components of mobile robot 1.

**Figure 8:**
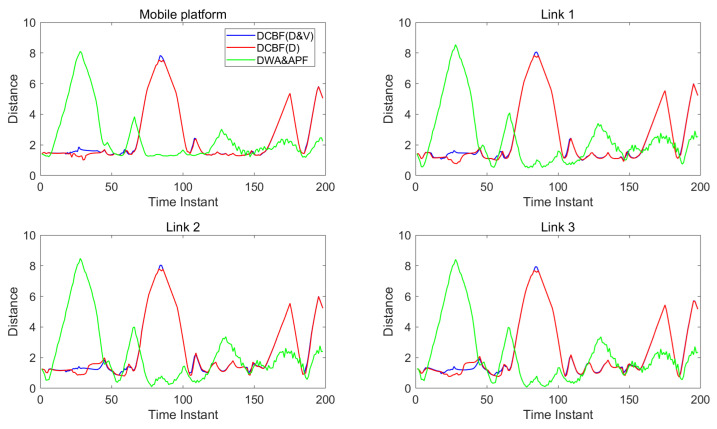
The distance (in meters) between humans and the components of mobile robot 2.

Figure 7 and Figure 8 show the distances between the two mobile robots and human units. The blue lines correspond to the control with DCBF constraints that consider both the closest point and most threatening point, designated as “DCBF (D&V)”. For comparison, the red lines correspond to the safe control with proximity-based DCBF constraints, designated “DCBF (D)”, and the green lines correspond to the control with DWA&APF method. It can be observed that with our method, all parts of the mobile robots maintain safe distances from humans. In contrast, targets to be dodged are incorrectly selected by proximity-based DCBF and undesired closer human–robot distances at subsequent moments occur, as proximity-based DCBF neglects the relative velocity during human–robot interactions and it treats humans as static obstacles at the current moment. Meanwhile, the mobile robots’ moving trajectories by the DWA&APF method sometimes violate the safety distance when encountering overly dense scenarios or aggressive maneuvers. Additionally, in dense environments, our method achieves a slight temporal advantage over the proximity-based DCBF constraints and less oscillation compared with that by DWA&APF method.

**Figure 9:**
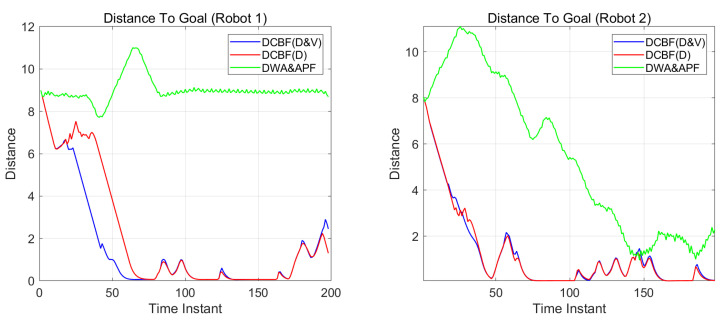
Distances to the goals (in meters) for the end effectors of the two mobile robots.

Figure 9 demonstrates the target convergence process of dual mobile robots’ end-effectors, where red and blue trajectories represent Robot 1 and 2, respectively. Comparative experiments reveal that under integrated constraints with proximity-based DCBF (36) and highest-threatening-index-based DCBF (37), Robot 1 reaches its target at the 60th time instant, converging 25% faster compared to the distance-constraint-only approach, which requires 80 time instants. Both configurations of constraints enable Robot 2 to synchronously arrive at its target at the 78th instant, limited by narrow passage dynamic constraints. Meanwhile, by means of the DWA&APF method, Robot 2 successfully initiates its navigation task. However, Robot 1 fails to move towards its target point. This is due to the fact that a human unit is near Robot 1 initially, causing the controller to prioritize obstacle avoidance. Subsequently, as Robot 2 approaches, it acts with other obstacles to obstruct Robot 1, leading Robot 1 to become stuck and unable to complete the navigation. Since Robot 1 is stuck near Robot 2’s target point, Robot 2 also fails to reach its destination. This performance enhancement stems from the proactive threat mitigation mechanism embedded in our proposed integrated constraints, which accelerates disengagement from hig y dense human–robot coexistence zones.

## 6. Conclusions

This paper establishes a safe control framework for environments with multiple coexisting mobile robots and humans. The coupled kinematic model for a mobile platform and a robot arm is firstly built with defined discrete-time control barrier function (DCBF) and discrete-time Lyapunov function (DCLF). Subsequently, the closest distances between parts of humans and the mobile robots are solved by convex programming with parametric description of pairs of skew lines. Specifically, the threatening index is defined and incorporated into DCBF by mutual projections of the relative velocity and common normal vector of skew line segments. Simulation of a scenario with two mobile robots and three humans validated the effectiveness of the proposed method. The proposed method can be applied to the safe control of mobile robots in crowded human–robot environments. The kinematic modeling of the current work is applicable to the mobile robot with Mecanum wheels. In future work, we will extend our proposed method to a mobile robot with power caster wheels and dual robotic arms.

## Data Availability

Data are contained within the article.

## Supplementary Materials

The following supporting information can be downloaded at: https://www.mdpi.com/article/10.3390/s25134005/s1.

## Abbreviations

The following abbreviations are used in this manuscript:

APF	Artificial Potential Field
CBF	Control Barrier Function
CSRL	CBF-based Safe Reinforcement Learning
DCBF	Discrete-time Control Barrier Function
DCLF	Discrete-time Control Lyapunov Function
DRL	Deep Reinforcement Learning
DWA	Dynamic Window Approach
GNN	Graph Neural Networks
MPC	Model Predictive Control
NBT	Neural Belief Tracking

## References

[1] Ehrmann C., Min J., Zhang W. (2024). Hig y flexible robotic manufacturing cell based on holistic real-time model-based control. Procedia CIRP.

[2] Stillström C., Jackson M. (2007). The concept of mobile manufacturing. J. Manuf. Syst..

[3] Villani V., Pini F., Leali F., Secchi C. (2018). Survey on human–robot collaboration in industrial settings: Safety, intuitive interfaces and applications. Mechatronics.

[4] Park D.H., Hoffmann H., Pastor P., Schaal S. (2008). Movement reproduction and obstacle avoidance with dynamic movement primitives and potential fields. Proceedings of the Humanoids 2008-8th IEEE-RAS International Conference on Humanoid Robots.

[5] Merkt W., Ivan V., Vijayakumar S. (2019). Continuous-time collision avoidance for trajectory optimization in dynamic environments. Proceedings of the 2019 IEEE/RSJ International Conference on Intelligent Robots and Systems (IROS).

[6] Lacevic B., Rocco P., Zanchettin A.M. (2013). Safety assessment and control of robotic manipulators using danger field. IEEE Trans. Robot..

[7] Ames A.D., Grizzle J.W., Tabuada P. (2014). Control barrier function based quadratic programs with application to adaptive cruise control. Proceedings of the 53rd IEEE Conference on Decision and Control.

[8] Taylor A.J., Ames A.D. (2020). Adaptive safety with control barrier functions. Proceedings of the 2020 American Control Conference (ACC).

[9] AbuJabal N., Baziyad M., Fareh R., Brahmi B., Rabie T., Bettayeb M. (2024). A comprehensive study of recent path-planning techniques in dynamic environments for autonomous robots. Sensors.

[10] Mrkšic N., Séaghdha D.O., Wen T.H., Thomson B., Young S. (2017). Neural belief tracker: Data-driven dialogue state tracking. arXiv.

[11] Zou A.M., Hou Z.G., Fu S.Y., Tan M. (2006). Neural networks for mobile robot navigation: A survey. Proceedings of the Advances in Neural Networks—ISNN 2006.

[12] Abdelrahman A.F., Valdenegro-Toro M., Bennewitz M., Plöger P.G. (2024). A neuromorphic approach to obstacle avoidance in robot manipulation. arXiv.

[13] Singh R., Ren J., Lin X. (2023). A review of deep reinforcement learning algorithms for mobile robot path planning. Vehicles.

[14] Hart F., Waltz M., Okhrin O. (2024). Two-step dynamic obstacle avoidance. Knowl.-Based Syst..

[15] Deng Y., Gao J., Feroskhan M. (2024). Ensuring safety in target pursuit control: A CBF-safe reinforcement learning approach. arXiv.

[16] Zhang Y., Tian G., Wen L., Yao X., Zhang L., Bing Z., He W., Knoll A. (2024). Online efficient safety-critical control for mobile robots in unknown dynamic multi-obstacle environments. arXiv.

[17] Li Z., Shi N., Zhao L., Zhang M. (2024). Deep reinforcement learning path planning and task allocation for multi-robot collaboration. Alex. Eng. J..

[18] Arents J., Abolins V., Judvaitis J., Vismanis O., Oraby A., Ozols K. (2021). Human–robot collaboration trends and safety aspects: A systematic review. J. Sens. Actuator Netw..

[19] Katona K., Neamah H.A., Korondi P. (2024). Obstacle avoidance and path planning methods for autonomous navigation of mobile robot. Sensors.

[20] Finean M.N., Petrović L., Merkt W., Havoutis I.M.I. (2023). Motion planning in dynamic environments using context-aware human trajectory prediction. Robot. Auton. Syst..

[21] Schneier M., Schneier M., Bostelman R. (2015). Literature Review of Mobile Robots for Manufacturing.

[22] Zhu Y., Chen S., Zhang C., Piao Z., Yang G. (2024). Development of adaptive safety constraint by predicting trajectories of closest points between human and co-robot. J. Intell. Manuf..

[23] Chen S., Zhu Y., Liu Y., Zhang C., Piao Z., Yang G. (2022). A “look-backward-and-forward” adaptation strategy for assessing parameter estimation error of human motion prediction model. IEEE Robot. Autom. Lett..

[24] Giallanza A., Scalia G.L., Micale R., Fata C.M.L. (2024). Occupational health and safety issues in human-robot collaboration: State of the art and open challenges. Saf. Sci..

[25] Han D., Park M.Y., Choi J., Shin H., Rhim S. (2021). Analysis of human-robot physical interaction at collision. Proceedings of the 2021 IEEE International Conference on Intelligence and Safety for Robotics (ISR).

[26] Koppenborg M., Nickel P., Naber B., Lungfiel A., Huelke M. (2017). Effects of movement speed and predictability in human–robot collaboration. Hum. Factors Ergon. Manuf. Serv. Ind..

[27] Ferraguti F., Landi C.T., Costi S., Bonfè M., Farsoni S., Secchi C., Fantuzzi C. (2020). Safety barrier functions and multi-camera tracking for human–robot shared environment. Robot. Auton. Syst..

[28] Merckaert K., Convens B., Wu C.j., Roncone A., Nicotra M.M., Vanderborght B. (2022). Real-time motion control of robotic manipulators for safe human–robot coexistence. Robot. Comput.-Integr. Manuf..

[29] Ames A.D., Xu X., Grizzle J.W., Tabuada P. (2017). Control Barrier Function Based Quadratic Programs for Safety Critical Systems. IEEE Trans. Autom. Control.

[30] Sabouni E., Ahmad H.M.S., Xiao W., Cassandras C.G., Li W. (2023). Optimal Control of Connected Automated Vehicles with Event-Triggered Control Barrier Functions: A Test Bed for Safe Optimal Merging. arXiv.

[31] Shi K., Chang J., Feng S., Fan Y., Wei Z., Hu G. (2024). Safe Human Dual-Robot Interaction Based on Control Barrier Functions and Cooperation Functions. IEEE Robot. Autom. Lett..

[32] Yang T., Miao Z., Yi G., Wang Y. Safety-Critical Control of Quadrotor UAVs with Control Barrier Functions. Proceedings of the 2022 IEEE International Conference on Robotics and Biomimetics (ROBIO).

[33] Xiao W., Belta C. (2022). High-Order Control Barrier Functions. IEEE Trans. Autom. Control.

[34] Liu J., Li M., Huang J.K., Grizzle J.W. (2023). Realtime Safety Control for Bipedal Robots to Avoid Multiple Obstacles via CLF-CBF Constraints. arXiv.

[35] van Wijk D.E.J., Coogan S., Molnar T.G., Majji M., Hobbs K.L. (2024). Disturbance-Robust Backup Control Barrier Functions: Safety Under Uncertain Dynamics. IEEE Control Syst. Lett..

[36] Min X., Baldi S., Shi Y., Yu W. (2025). Safe Control of Multi-Agent Systems via Low-Complexity Control Barrier Functions. IEEE Trans. Autom. Control.

[37] Buyukkocak A.T., Aksaray D., Yazıcıoğlu Y. Control Barrier Functions with Actuation Constraints under Signal Temporal Logic Specifications. Proceedings of the 2022 European Control Conference (ECC).

[38] Emam Y., Notomista G., Glotfelter P., Kira Z., Egerstedt M. (2022). Safe Reinforcement Learning Using Robust Control Barrier Functions. arXiv.

[39] Garg K., Usevitch J., Breeden J., Black M., Agrawal D., Parwana H., Panagou D. (2024). Advances in the Theory of Control Barrier Functions: Addressing practical challenges in safe control synthesis for autonomous and robotic systems. Annu. Rev. Control.

[40] Qin W., Yi H., Fan Z., Zhao J. (2025). Haptic Shared Control Framework with Interaction Force Constraint Based on Control Barrier Function for Teleoperation. Sensors.

[41] Cao L., Tang L., Cao S., Sun Q., Zhou G. (2025). Smooth Optimised A*-Guided DWA for Mobile Robot Path Planning. Appl. Sci..

