# Supplementary figures and images for: Revised Control Barrier Function with Sensing of Threats from Relative Velocity Between Humans and Mobile Robots
Source: Sensors (Basel). 2025 Jun 27;25(13):4005. doi: 10.3390/s25134005 (PMC12251740; doi:10.3390/s25134005)

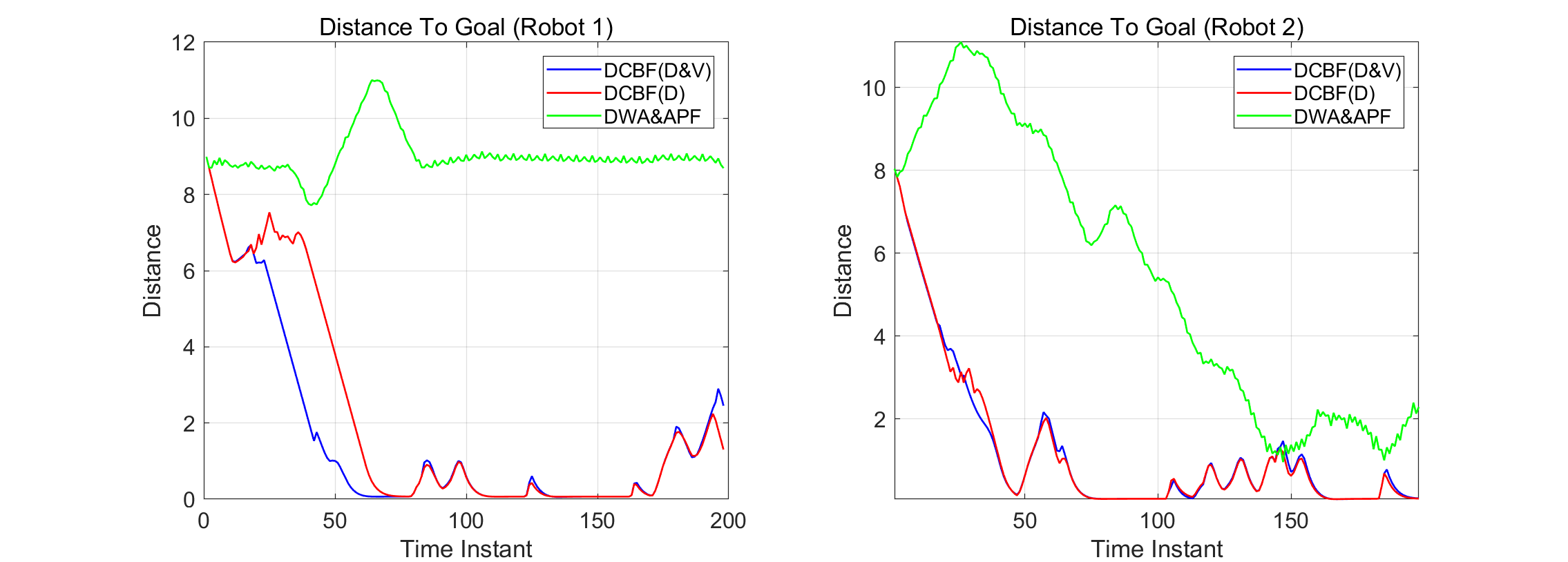

Supplement: Supplementary file 1 [file sensors-25-04005-s001.zip › code/plotdata/goal_compare_all.png]

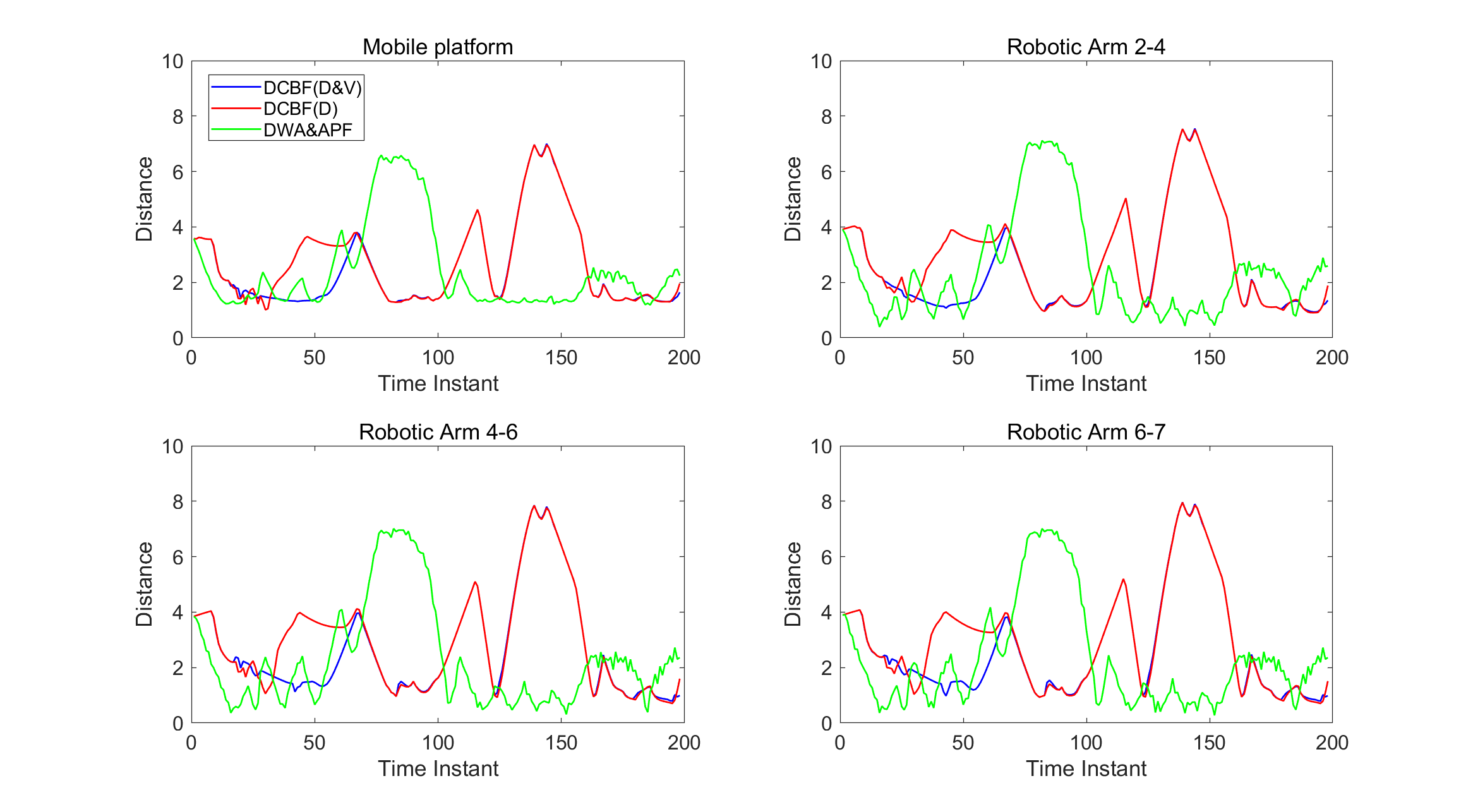

Supplement: Supplementary file 1 [file sensors-25-04005-s001.zip › code/plotdata/robot1_compare_all.png]

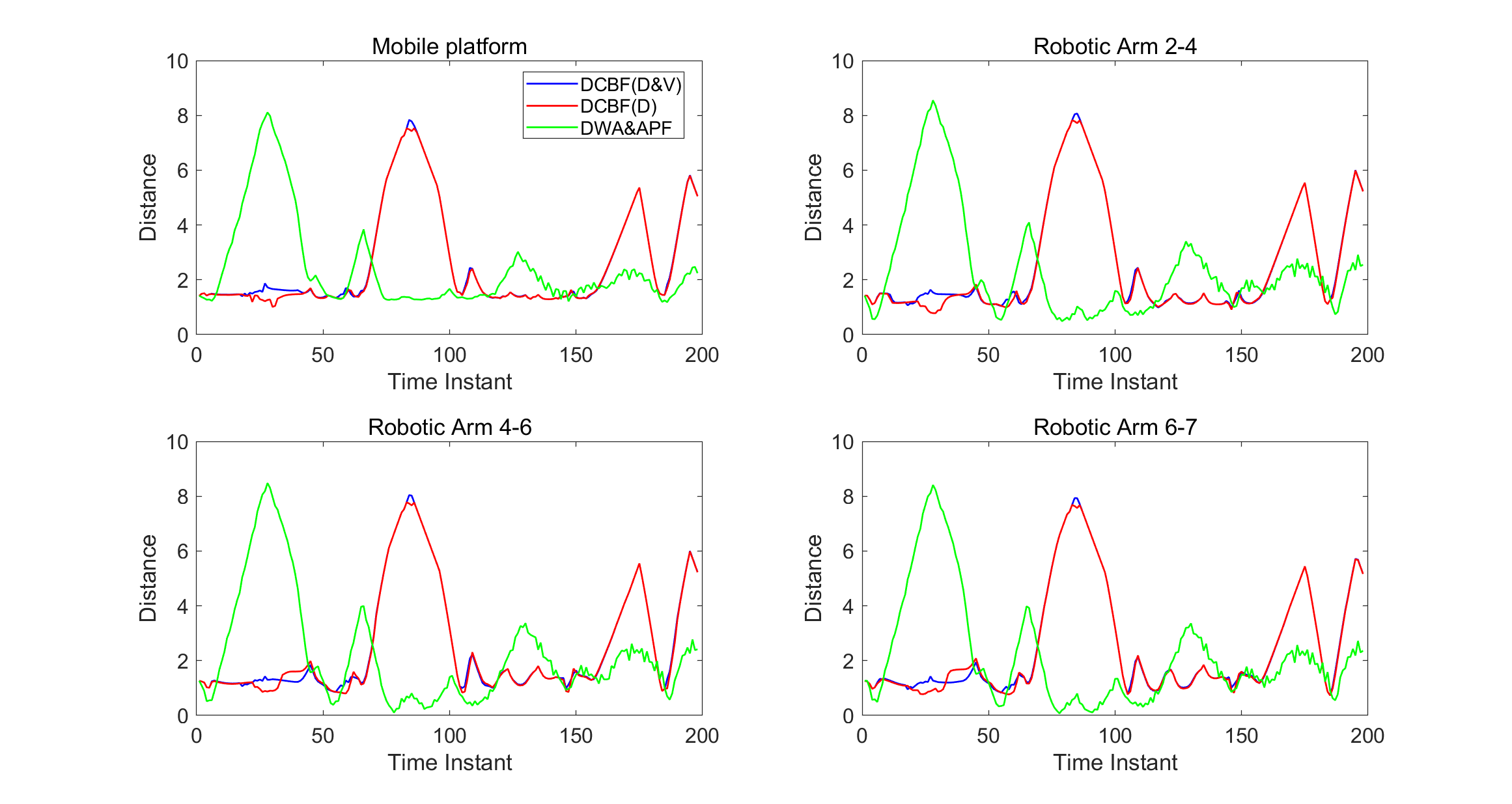

Supplement: Supplementary file 1 [file sensors-25-04005-s001.zip › code/plotdata/robot2_compare_all.png]
